# WARRIOR-trial - is routine radiography following the 2-week initial follow-up in trauma patients with wrist and ankle fractures necessary: study protocol for a randomized controlled trial

**DOI:** 10.1186/s13063-015-0600-x

**Published:** 2015-02-27

**Authors:** Nikki L Weil, M Frank Termaat, Sidney M Rubinstein, Mostafa El Moumni, Wietse P Zuidema, Robert Jan Derksen, Pieta Krijnen, Leti van Bodegom-Vos, Klaus W Wendt, Cornelis van Kuijk, Frits R Rosendaal, Roelf S Breederveld, J Carel Goslings, Inger B Schipper, Maurits W van Tulder

**Affiliations:** Department of Surgery/Trauma Surgery, Leiden University Medical Centre, P.O. Box 9600, 2300 RC Leiden, the Netherlands; Department of Health Sciences, VU University Amsterdam, de Boelelaan 1085, 1081 HV Amsterdam, the Netherlands; Department of Surgery, University Medical Centre Groningen, P.O. Box 30.001, 9700 RB Groningen, the Netherlands; Department of Surgery/Trauma Surgery, VU University Medical Centre Amsterdam, P.O. Box 7057, 1007 MB Amsterdam, the Netherlands; Department of Surgery, Red Cross Hospital, P.O. Box 1074, 1940 EB Beverwijk, the Netherlands; Department of Medical Decision Making, Leiden University Medical Centre, P.O. Box 9600, 2300 RC Leiden, the Netherlands; Department of Radiology and Nuclear Medicine, VU University Medical Centre Amsterdam, P.O. Box 7057, 1007 MB Amsterdam, the Netherlands; Department of Clinical Epidemiology, Leiden University Medical Centre, P.O. Box 9600, 2300 RC Leiden, the Netherlands; Trauma Unit, Academic Medical Centre, P.O. Box 22660, 1100 DD Amsterdam, the Netherlands; Department of Health Sciences and the EMGO-Institute, VU University, de Boelelaan 1085, 1081 HV Amsterdam, the Netherlands

**Keywords:** Wrist fracture, Distal radius fracture, Ankle fracture, Radiography, Functional outcome, Cost-effectiveness, Routine follow-up

## Abstract

**Background:**

Extremity fractures such as wrist and ankle fractures are a common and costly healthcare problem. The management of these fractures depends on fracture type and loss of congruity of the joint, resulting in cast immobilization or operative treatment. Loss of congruity or displacement leading to uneven joint loading, osteoarthritis and an increased probability of a poor functional outcome should be identified within the first 2 weeks post-trauma, based upon radiographs to determine optimal treatment. After this period, routine radiographs are scheduled for monitoring the bone-healing process. Current protocols describe imaging at 1, 2, 6 and 12 weeks post-trauma. However, it is questionable whether routine radiography following the initial follow-up ( 2-weeks post-trauma) is cost effective.

The aim of this study is to determine whether a modification of the radiographic follow-up protocol can be conducted with no worse outcome and less cost than the current standard of care for patients with a wrist or ankle fracture.

**Methods/design:**

In a multicenter randomized controlled trial, 697 patients aged 18 years or older will be included: 385 wrist fracture- and 312 ankle fracture patients. Patients will be randomized into two groups: Group 1 receives usual care, consisting of radiographs 1, 2, 6 and 12 weeks post-trauma; Group 2 receives radiographs beyond the initial follow-up only when clinically indicated. The primary outcome is the overall extremity-specific function. For wrist fractures, this includes the Disabilities of the Arm, Shoulder and Hand Score; for the ankle fractures, this includes the Olerud and Molander ankle score. Secondary outcomes include: healthcare cost, the specific function measured with the Patient Rated Wrist and Hand Evaluation for wrist fractures and American Academy of Orthopaedic Surgeons foot and ankle questionnaire for ankle fractures, pain-intensity, health-related quality of life, self-perceived recovery, and complications. Both groups will be monitored at 1, 2, and 6 weeks and 3, 6, and 12 months.

**Discussion:**

This study will provide data on (cost) effectiveness of routine radiography in the follow-up of wrist and ankle fractures, and could pave the way for a change in (inter)national protocols.

**Trial registration:**

Netherlands Trial Register NTR4610, registration date 22 June 2014.

## Background

Extremity fractures are a common and costly healthcare problem affecting all age groups [1]. In the adult population, the incidence of fractures to the wrist (distal radius) and ankle (malleolar fractures) in the Netherlands is about 20,000 and 15,000 per year, respectively [2]. These are the most common fractures encountered, accounting for about 18% and 10% of all fractures, respectively. Treatment of fractures consists of either immobilization or surgical fixation (18% of distal radius fractures and 35% of ankle fractures) [3]. The decision to operate should preferably be established within the first 2 weeks, as after this period the bone healing has advanced to a stage in which surgical treatment is more difficult. Loss of congruity or (secondary) displacement can lead to uneven joint loading, osteoarthritis and a poor outcome [4]. Resolution of soft-tissue swelling and poor cast application leaves patients at increased risk of fracture displacement [5]. This risk makes early identification of the loss of congruity or (secondary) displacement paramount. Displacement can be identified within the first 2 weeks based upon radiographic imaging. After this period, regular radiographs and clinical assessment are frequently scheduled, based upon existing local trauma protocols for monitoring the fracture-healing process and clinical outcomes ([6,7] and web-based protocols listed at the end of the article).

### Current follow-up protocols

Current protocols that are used both nationally and internationally recommend radiographic assessment at all routine visits (that is, 1, 2, 6 and 12 weeks post-trauma) in order to monitor fracture healing in fractures of the distal radius and ankle. However, it is questionable whether routine imaging following the initial follow-up (that is, post 2-weeks) is justified [8]. Justification for obtaining radiographs beyond the initial follow-up includes documentation of splint application, resident education, assurance that the fracture has not displaced, and medico-legal protection against claims of unintended harm [8]. Three retrospective studies have suggested that routine radiography performed beyond the 2-week follow-up results in longer waiting room visits, unnecessary radiation exposure and increased healthcare costs without providing additional clinical information that impacts clinical decision-making [8-10]. Thus, there is evidence from multiple sources that routine radiographic imaging beyond the 2-week period is clinically redundant. Hence, the current protocol would not appear to be cost effective. In The Netherlands, about 35,000 adults present yearly with fractures of the distal radius and ankle. Based upon the current costs of imaging [11], it is estimated that in the Netherlands approximately €2.9 million could be saved yearly by modifying the current imaging protocol.

### Rationale

We hypothesize that a modification of the current radiographic follow-up protocol towards a protocol with fewer radiographs for patients with distal radius or ankle fractures will lead to significant cost savings without compromising quality of care. The aim of this study is to determine whether a modification of the radiographic follow-up protocol (no standard routine radiographs after 2-week initial follow-up) can be conducted for less direct and indirect healthcare costs, but with no worse outcomes than the current standard of care prescribed for patients with distal radius or ankle fractures.

## Methods/design

### Study design

The WARRIOR-trial (Wrist and Ankle fractures Routine RadIOgraphy Reduction) is a multicenter randomized controlled trial using a non-inferiority design.

The trial includes two study populations: distal radius fracture patients and ankle fracture patients. The interventional effects will be analyzed separately for each type of fracture as independent studies. The same design and research questions apply for both studies.

The study will be conducted in three academic hospitals in The Netherlands (Leiden University Medical Center (LUMC), VU University Medical Center Amsterdam (VUmc) and University Medical Center Groningen(UMCG)). This study is approved by the Medical Ethics Committee LUMC for all participating centers (Number P14.086). The trial is registered in the Dutch Trial Register (Nederlands Trial Register NTR4610, registration date 22 June 2014). Study results will be reported following the Consolidation of Standards of Reporting Trials guidelines [12,13].

### Study population

All patients presenting to the emergency department with a distal radius or ankle fracture and meeting the following criteria are eligible for inclusion: men or women aged 18 years or older; fracture of the distal radius (Orthopaedic Trauma Association Committee for Coding and Classification classification type A-C [14]) or fracture of the ankle (Lauge-Hansen classification: supination-adduction stage II, supination-external rotation stage II-IV, pronation-external rotation stage I-IV [15]); sufficient understanding of the Dutch language in order to independently complete the follow-up questionnaires; written informed consent provided by the patient.

Patients meeting one or more of the following criteria will be excluded: pathological fractures; complicated fractures (Gustilo grade 2 and 3); multi-extremity fractures; psychiatric conditions; unable to complete follow-up (for example, patients living abroad, incarcerated subjects).

### Study procedures

The inclusion and exclusion criteria will be checked for all patients presenting with distal radius or ankle fractures in the emergency department of the participating centers. Eligible patients will be informed of the aims and design of the study and will receive written information.

At the subsequent visit (that is, within the first 2 weeks after the initial visit) the local investigator or research nurse will check the inclusion and exclusion criteria once more, provide additional information, answer any questions regarding the study and obtain written informed consent.

### Randomization

Randomization of patients will be carried out after obtaining written informed consent using an online randomization program (ProMISe, www.clinicalresearch.nl). Randomization will be stratified by center, type of fracture (distal radius or ankle) and treatment (conservative or surgical). Patients will be randomized into the following two groups.

#### Group 1 – usual care

Group 1 will receive usual care according to the current national protocol, indicating clinical follow-up as well as radiographic evaluations in the outpatient clinic at 1, 2, 6, and 12 weeks after trauma.

#### Group 2 – reduced imaging

Group 2 will receive the same clinical evaluations as the usual care group; however, no routine radiographs will be performed beyond the initial 2 weeks. Radiography during follow-up beyond the initial 2 weeks will be allowed if any of the following are present: new trauma to the distal radius or ankle; pain score >6 on the 11-point visual analogue scale (VAS); loss of range of motion (ROM); neurovascular symptoms; at the discretion of the clinician, in which case the indication for radiography is to be recorded in the medical records.

Aftercare apart from radiology will be identical for both study groups and according to the local standards.

At 6 and 12 weeks, 6 months and 1 year, patients will be requested to fill out the questionnaires. The patient can chose between a paper version or a web-based version of the questionnaire. Reminders will be sent when necessary, and attempts will be made to track drop-outs if possible. An overview of follow-up and measurements is provided in Table 1.

**Table 1:** **Flowsheet**

	**ER**	**Week 1**	**Week 2**	**Week 6**	**Week 12**	**Week 26**	**Week 52**
Written patient information	X						
Randomization			X				
Baseline characteristics			X				
Radiographs							
Group 1		X*	X*	X	X	○	○
Group 2		X*	X*	○	○	○	○
Visual analogue score			X	X	X	X	X
Function scores (OMAS/AAOS or DASH/PRWHE)			X	X	X	X	X
Quality of Life (SF-36/EQ5D)			X	X	X	X	X
Self-perceived recovery				X	X	X	X
Direct and indirect healthcare costs				X	X	X	X
ROM				X	X		
Complications		X*	X*	X	X	X	X

*Standard Protocol first 2 weeks. X, Measurement collected at each time point; ○, radiography only when clinically indicated; AAOS, American Academy of Orthopaedic Surgeons; DASH, Disabilities of the Arm, Shoulder and Hand Score; EQ5D, EuroQol-5D; ER, emergency room; OMAS, Olerud and Molander Ankle Score; PRWHE, Patient-Rated Wrist and Hand Evaluation; ROM, range of motion; SF-36, Short form-36.

Subjects can leave the study at any time for any reason if they wish to do so without any consequences; patients randomized to Group 2 will revert to the usual care, the care according to the current protocol, after leaving the study.

### Outcomes

#### Primary

The primary outcome is the overall extremity-specific functional status, which for both types of fracture will be measured using Dutch versions of the following questionnaires.

For distal radius fractures the Disabilities of the Arm, Shoulder and Hand Score (DASH) will be used. DASH is the most commonly used questionnaire for measuring upper extremity function [16]. It is a 30-item questionnaire that was designed to evaluate symptoms and physical function in patients with upper extremity disorders. Scoring is done by subtracting 30 points from the sum of the responses. This number is then divided by 1.2 to obtain the score. Missing items are replaced by the mean value of the responses before summing. Scores range from 0 to 100, with 0 reflecting no pain/disability [16-18]. The overall score cannot be calculated if three items or more are missing [16,17,19].

For ankle fractures the Olerud and Molander Ankle Score (OMAS) will be used. The OMAS is a nine-item questionnaire evaluating the functional outcome of the ankle after injury. Scores range from 0 to 100, with 100 reflecting no pain/disability [20].

Secondary outcome measures are: costs; specific function measured with the Patient-Rated Wrist and Hand Evaluation (PRWHE) for the wrist fractures and the American Academy of Orthopaedic Surgeons (AAOS) foot and ankle questionnaire for ankle fractures; pain intensity at rest; health-related quality of life measured with the Short Form-36 (SF-36) questionnaire and the EuroQol-5D (EQ5D) questionnaire; self-perceived recovery; ROM; and complications.

Costs will be investigated using a healthcare utilization questionnaire. The costs of healthcare consumption include, for example, costs for consulting the general practitioner, company doctor and/or physiotherapist, costs of medication and hospitalization, and costs of absence from work associated with the fracture of the distal radius or ankle.

The PRWHE is a 15-item (five pain and 10 disability items) questionnaire that rates pain and disability of the wrist in functional activities [18]. Scoring is done by adding up the sum of the five pain items and the sum of the 10 disability items, and dividing by two. Missing items are replaced by the mean score of the subscale. Scores range from 0 to 100, with 0 reflecting no pain/disability [16-18].

The AAOS foot and ankle questionnaire is a commonly used 25-item questionnaire that combines items on pain, function, stiffness and swelling, and allows assessment of the patient’s perception of the condition of the ankle/foot and the treatment progress [21,22]. Scoring is done by an online worksheet that can be found at http://www.aaos.org/research/outcomes/outcomes_lower.asp.

Pain intensity at rest is measured using a continues VAS, where 0 reflects no pain and 10 the worst possible pain.

The SF-36 is a 36-item questionnaire about health status recorded by the patient. The items are subdivided into eight health concepts. Combining these concepts leads to two health scales: a physical health scale and a mental health scale [23,24]. The scores range from 0 to 100 points, with higher scores reflecting better function. The derived score has to be converted to a norm-based score in order to make it possible to compare the score with the norms for the general population of the United States (1998), in which each scale was scored to have the same average of 50 points and the same standard deviation of 10 points [25,26].

The EQ5D is a five-item questionnaire to assess health-related quality of life. The questionnaire measures the ability to walk, the ability to perform daily activities, depression, anxiety and pain on a three-point scale. These scores are combined to describe the health status of the patient, which can be linked to a preference (utility) for the health status obtained from the general population [27,28].

The self-perceived recovery is measured on a five-point Likert-scale.

The ROM of the wrist or ankle joint will be measured during the outpatient clinic visits at weeks 6 and 12 after trauma. The ROM in the wrist will be tested by measuring the palmar flexion, dorsal extension, pronation and supination. The ROM in the ankle will be tested by measuring the flexion, plantar flexion, inversion and eversion.

Complications that will be registered include infection, nonunion, malunion and implant failure. These will be recorded from the medical charts by the investigator.

Potentially confounding variables, such as age, gender and socio-demographic characteristics will be registered.

### Sample size calculation

For both types of fractures (distal radius and ankle), the sample size was determined separately for both patient groups. Based upon a margin of non-inferiority, which is often the smallest value with a clinically important effect [29], of 9 points on the DASH and OMAS (which is based upon what is assumed to be clinically significant [30]) and a standard deviation of 14 [31] and 20 [32], respectively, 70 distal radius and 142 ankle fractures are necessary to demonstrate non-inferiority (power 0.85, alpha 0.05).

To enable a sensitivity analysis for evaluating the study outcomes for two relevant subgroups (that is, conservatively and surgically treated patients), more patients are needed. Based on the empirical treatment ratio for conservative:surgical of 8:2 for distal radius fractures and 5:5 for ankle fractures, we calculated that 142/0.5 = 284 patients with ankle fractures and 70/0.2 = 350 patients with distal radius fractures will need to be included. Accounting for a 10% loss to follow-up means 312 patients with an ankle fracture and 385 patients with a distal radius fracture are to be recruited.

### Statistical analysis

Data will be analyzed using the IBM SPSS version 20 or higher (Armonk, NY: IBM Corp.). Characteristics of the patients in the treatment groups will be presented using descriptive statistics (mean ± standard deviation, median (range) or proportion) to assess if balanced groups were obtained after randomization.

The data analysis will be conducted according to the intention-to-treat principle, with a normal approximation for all questionnaires at 6, 12, 26 and 52 weeks for both types of fractures. For analysis of the primary study outcome, linear and generalized multilevel analyses will be used accounting for dependency within hospitals, clinicians and patients over time (four-level model). This means, among other things, that the data are to be analyzed longitudinally and, thus, the follow-up measurements are to be incorporated in the analyses. Results will be expressed as confidence intervals and these will be subsequently compared to the margin of non-inferiority in order to make inferences about the non-inferiority of the new intervention strategy. In addition to crude analyses, all analyses will also be adjusted for pre-defined prognostic factors, such as age, gender and fracture type. In a secondary analysis, a per-protocol analysis will be conducted which will account for ‘contamination’, meaning those randomized to the intervention group but nonetheless requiring follow-up imaging not allowed in the original treatment design (because there was a clinical indication for imaging).

The secondary clinical outcome measures of quality of life and pain will be analyzed in the same way as the primary outcome measure. The secondary outcome measures of ROM and complications will be compared between the ‘usual care’ group and the ‘reduced images’ group over time.

The cost questionnaires will be used to perform an economic evaluation, which will be conducted from a societal perspective alongside the randomized controlled trial. The aim of the economic evaluation is to measure, value and analyze total costs of patients with distal radius and ankle fractures and to relate the difference in costs to the difference in effects between the intervention group which will receive only the initial follow-up radiographs (at 1 and 2 weeks) and the comparison (that is, usual care) which will receive imaging at the initial follow-up plus at 6 and 12 weeks. Both cost-effectiveness and cost-utility analysis will be performed. The time horizon of the economic evaluation is 12 months. Sensitivity analyses will be performed to assess the robustness of the results using different assumptions regarding costs and effects.

The underlying assumption surrounding missing data for this study is that the data are missing at random. Therefore, given the mixed model approach for the longitudinal analyses, missing data will not be imputed; however, we will correct for baseline covariates thought to be associated with the outcome, such as age, gender, and variables related to the condition (for example, fracture type), as well as variables thought to be related to missingness. In a sensitivity analysis, complete data sets will be compared to incomplete data sets in order to determine how robust these findings are.

### Ethical considerations

The study design is in accordance with the Declaration of Helsinki, version Fortaleza (Brazil), October 2013 [33], and in accordance with the Dutch Medical Research Involving Human Subjects Act. Patients will not experience any benefit from participating in the study. No risk is involved for the group receiving usual care (Group 1). In Group 2 without standard radiographs after 2 weeks, the risk is considered minimal because radiographs will be made when clinically indicated.

This study is approved by the medical research ethics committee for all participating centers. The Medical Ethics Committee LUMC acts as central ethics committee for this trial (Number P14.086). An insurance which is in accordance with the legal requirements in the Netherlands (Article 7 WMO and the Measure regarding Compulsory Insurance for Clinical Research in Humans of 23 June 2003) has been obtained. This insurance provides cover for damage to research subjects through injury or death caused by the study. Once a year, information will be provided to the medical research ethics committee on the numbers of subjects included and numbers of subjects that have completed the trial, serious adverse events/serious adverse reactions, and other problems.

## Discussion

The current distal radius and ankle fracture protocols recommend radiographic assessment at all routine visits. It is questionable whether routine imaging following the initial follow-up (that is, after 2 weeks), after which definitive treatment is established (being surgical or conservative), is justified [8]. Three retrospective studies have suggested that radiographs performed beyond the 2-week follow-up result in longer waiting room visits, unnecessary radiation exposure and increased healthcare costs without providing additional clinical information which impacts clinical decision-making [8-10]. Alongside the clinical question about the justification of the radiographs after the initial follow-up period, a cost-effectiveness analysis is indicated as the current protocols appear not to be cost effective. A randomized controlled trial is needed to investigate whether a reduced imaging protocol can be conducted for less cost, but with no worse outcome than the current standard of care in those with distal radius and ankle fractures. This study will provide necessary data on (cost) effectiveness of routine radiography in the follow-up of distal radius and ankle fractures and could pave the way for a change in (inter)national protocols.

## Trial status

The trial started in July 2014, and is currently enrolling participants.

## Web based protocols

http://www.info-med.nl/Traumatologie.htm

http://www.inter-med.nl/heelkunde/traumaprotocol.htm

https://www.lumc.nl/org/traumacentrum-west/kenniscentrum/121031022731411/

## Abbreviations

AAOS: American Academy of Orthopaedic Surgeons
DASH: Disabilities of the Arm, Shoulder and Hand Score
EQ5D: EuroQol-5D
LUMC: Leiden University Medical Center
OMAS: Olerud and Molander Ankle Score
PRWHE: Patient-Rated Wrist and Hand Evaluation
ROM: Range of motion
SF-36: Short Form-36
UMCG: University Medical Center Groningen
VAS: Visual analogue scale
Vumc: VU University Medical Center
WARRIOR: Wrist and Ankle fractures Routine RadIOgraphy Reduction
